# Multiple Sources of Infection and Potential Endemic Characteristics of the Large Outbreak of Dengue in Guangdong in 2014

**DOI:** 10.1038/srep16913

**Published:** 2015-11-23

**Authors:** Shu-Qun Shen, Hai-Xia Wei, Yong-Hang Fu, Hao Zhang, Qing-Yi Mo, Xiao-Jun Wang, Sheng-Qun Deng, Wei Zhao, Yu Liu, Xiao-Shuang Feng, Wei Chen, Hong-Juan Peng

**Affiliations:** 1 Guangdong Provincial Key Laboratory of Tropical Disease Research, and Key Laboratory of Prevention and Control for Emerging Infectious Diseases of Guangdong Higher Institutes, School of Public Health and Tropical Medicine, Southern Medical University, #1023 South Shatai Road, Guangzhou, Guangdong Province, 510515, China; 2 Department of Clinical Laboratory, the 458th Hospital of PLA, #801 Dongfeng Dong Road, Guangzhou, Guangdong Province, 510602, China; 3 Guangdong Provincial Key Laboratory of Viral Hepatitis Research, Department of Infectious Diseases, Nanfang Hospital, Southern Medical University; 4 Department of pediatrics, Zhongshan Boai Hospital Affiliated to Southern Medical University, Zhongshan, Guangdong Province, 528403, China; 5 Max-Delbrueck-Center for Molecular Medicine. Robert-Roessle-str. 10, Berlin, 13125, Germany

## Abstract

A large outbreak of dengue, with the most documented cases, occurred in Guangdong China in 2014. Epidemiological studies and phylogenetic analysis of the isolated dengue virus (DENV) showed this outbreak was attributed to multiple sources and caused by at least two genotypes of DENV-1 (Genotypes I and III) and two genotypes of DENV-2 (Cosmopolitan and Asian I Genotypes). A retrospective review and phylogenetic analysis of DENV isolated in Guangdong showed that DENV-1 Genotype I strains were reported continuously during 2004–2014, Genotype III strains were reported during 2009–2014 ; DENV-2 Cosmopolitan and Asian I Genotype strains were reported continuously during 2012–2014. At least 45,171 cases were reported in this outbreak, with 65.9% of the patients in the 21–55-year-old group. A trend toward a decrease in the daily newly emerged cases lagged by approximately 20 days compared with the mosquito density curve. Several epidemiological characteristics of this outbreak and the stably sustained serotypes and genotypes of DENV isolated in Guangdong suggest that Guangdong has been facing a threat of transforming from a dengue epidemic area to an endemic area. The high temperature, drenching rain, rapid urbanization, and pandemic of dengue in Southeast Asia may have contributed to this large outbreak of dengue.

Dengue, a febrile viral disease transmitted by *Aedes* mosquitoes, is estimated to afflict 390 million people per year, 96 million of whom present diseases of differing severity, from asymptomatic infection to Dengue Hemorrhagic Fever (DHF) and Dengue Shock Syndrome (DSS)^1^. It has also been estimated that approximately 3900 million people in 128 countries are at risk of infection with dengue viruses^2^. The causative agent of dengue is dengue virus (DENV), which belongs to the family Flaviviridae and is to date classified into five serotypes (DENV−1, −2, −3, −4 and −5); all serotypes can classically cause undifferentiated fever^3^,^4^. In recent decades, the incidence and geographic expansion of dengue have grown dramatically worldwide, and the reasons are complex, including climate change, virus evolution, deteriorating vector control, increasing population mobility, and uncontrolled urbanization^5^,^6^,^7^,^8^.

In China, since the first confirmation of a DENV-4 epidemic in Foshan, Guangdong Province in 1978, dengue outbreaks have been reported in Guangdong, Hainan, Guangxi, Fujian, Zhejiang and Yunnan provinces^9^. Guangdong Province has the highest incidence of dengue in China, with cases being reported every year since 1997^9^,^10^,^11^, and is characterized by hot and humid subtropical weather; the summer is long, lasting from May to October, and the average annual rainfall is 1,700 mm^10^. Although dengue infections or outbreaks involving 4 serotypes have been reported in Guangdong in the past 30 years, DENV-1 has become the predominant cause of epidemics since 1991^9^,^11^. Recently, the dengue epidemic pattern observed in Guangdong has shown characteristics of a hypo-endemic nature: circulation of DENV-1 over consecutive years, young age groups being at a greater risk of infection, transition from a monotypic to a multitypic circulation (in 2010 and 2012, all 4 serotypes were detected one after another in indigenous outbreak locations), a higher seroprevalence of DENV antibodies in local inhabitants compared to that observed in non-endemic regions, and approximately 90.11% of dengue cases being indigenous^11^.

*Ae. albopictus*, a peridomestic mosquito that is known to be the major vector of dengue in Guangdong Province^12^,^13^, is displacing the fully domestic dengue vector *Ae. aegypti*^14^. Indeed, Guangdong has rapidly urbanized in the past 2–3 decades, and urbanization substantially increases the density, larval development and adult survival of *Ae. albopictus*, which in turn increases vector capacity and dengue transmission ability^15^.

## Results

### Description of the outbreak

**Figure 1:**
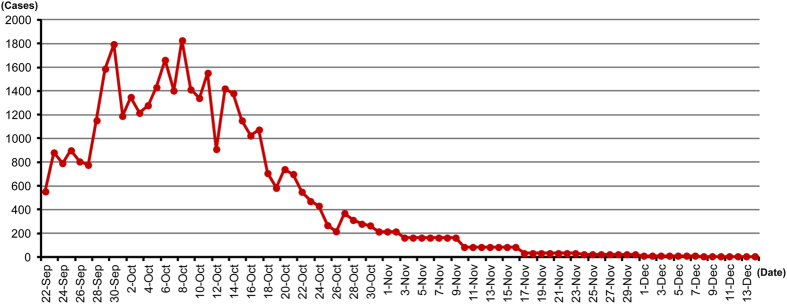
Daily newly emerged cases of dengue in Guangdong Province in 2014. After 12 cases of dengue were reported by June 11, 2014, an outbreak of dengue spread quickly throughout the entire province. The total number of cases of dengue was 45,171, with cases occurring until December 14, 2014. The daily newly emerged cases of dengue reached a peak between September 28 and October 17, exceeding 1000 cases each day, and then markedly decreased quickly between October 17 and December 8, 2014, to fewer than 44 cases a week.

Until June 11, 2014, only 12 cases of dengue had been reported to the Guangdong provincial Center for Diseases Control (CDC), including one case of indigenous dengue and 11 cases of imported dengue. Based on the 12 cases as of June 11 to the 45,171 cases reported by December 15, the outbreak lasted for more than 7 months, resulting in 6 deaths, even though 99.1% of the cases presented with mild symptoms. The daily newly emerged cases peaked between September 28 and October 17, accounting for 58% of all cases reported until December 15, 2014, with more than 1000 new cases reported every day (Fig. 1). After the peak, the daily new cases decreased quickly to a very low level in mid-November and then tapered off in the next month (Fig. 1).

### Clinical/laboratory diagnosis and manifestations

We sampled 200 clinically diagnosed and 2050 laboratory-confirmed Dengue Fever (DF) cases from the three hospitals in Guangdong Province. The symptom composition was as follows: fever (100%), headache (35.0%), rash (29.5%), myalgia (60.5%), hemorrhage (bleeding spots under the skin) (20.0%), bleeding (7.0%), arthralgia (18.0%), and fever + headache + rash + myalgia (6.9%). Among the laboratory-confirmed cases, approximately 56.2% exhibited leucopenia, 61.4% presented thrombocytopenia, and 76.8% and 42.6% showed serum ALT and AST elevation, respectively (Table 1). Among the 2050 laboratory diagnosed cases, 787 were PCR positive, 1286 NS1 antigen positive, 60 IgM positive, 17 IgG positive; and 33 were virus isolation positive. As these cases were diagnosed with only one method or combined more than one methods, the data may present an diagnosis efficiency among these diagnosis methods.

**Figure 2:**
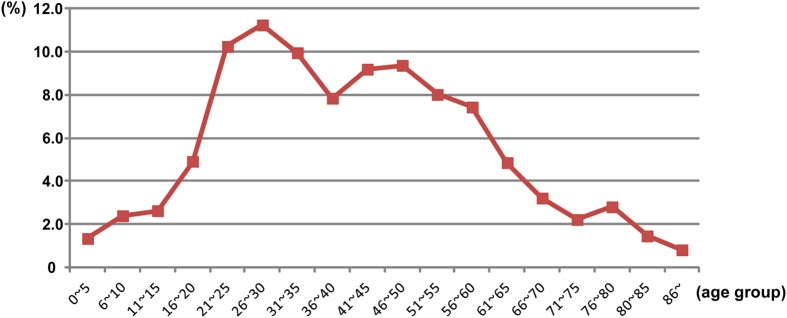
Age composition of the cases reported in the dengue outbreak in Guangdong in 2014. Patients aged 16–65 years old accounted for approximately 83.1% of all DF cases, with 65.9% in young age groups (21–55 years old). A main peak appeared in the 21- to 35-year-old group, and a secondary peak appeared in the 41- to 55-year-old group.

The DF cases consisted of 49.9% male patients. The median age was 40 years (range: 7 days to 92 years). Approximately 65.9% of patients were in the 21–55 age group (Fig. 2).

### Virus identification

Thirty-three dengue virus isolates were successfully isolated from the serum of different patients in the acute phase (within 5 days of disease onset) after inoculation and culture of baby hamster kidney (BHK-21) cells and named GZ2014-08, GZ2014-11, GZ2014-42, GZ2014-39, and GZ2014-B01 to B29. These isolates were identified by reverse transcription polymerase chain reaction (RT-PCR) followed by sequencing of the E-protein gene, and the sequences were submitted to GenBank under the following accession numbers: GZ2014-08 (gb|KP780169), GZ2014-11 (gb|KP780170), GZ2014-42 (gb|KP780171), GZ2014-B01 to B26 (gb|KT232178 to KT232203), and these 29 strains were identified as serotype 1; GZ2014-39 (gb|KP780172), GZ2014-B27 to B29 (gb|KT232204 to KT232206), and these 4 strains were confirmed as serotype 2.

### Phylogenetic analysis of isolated dengue viruses along with homologous strains reported in Guangdong and a retrospective review of the serotypes of dengue virus reported in Guangdong

**Figure 3:**
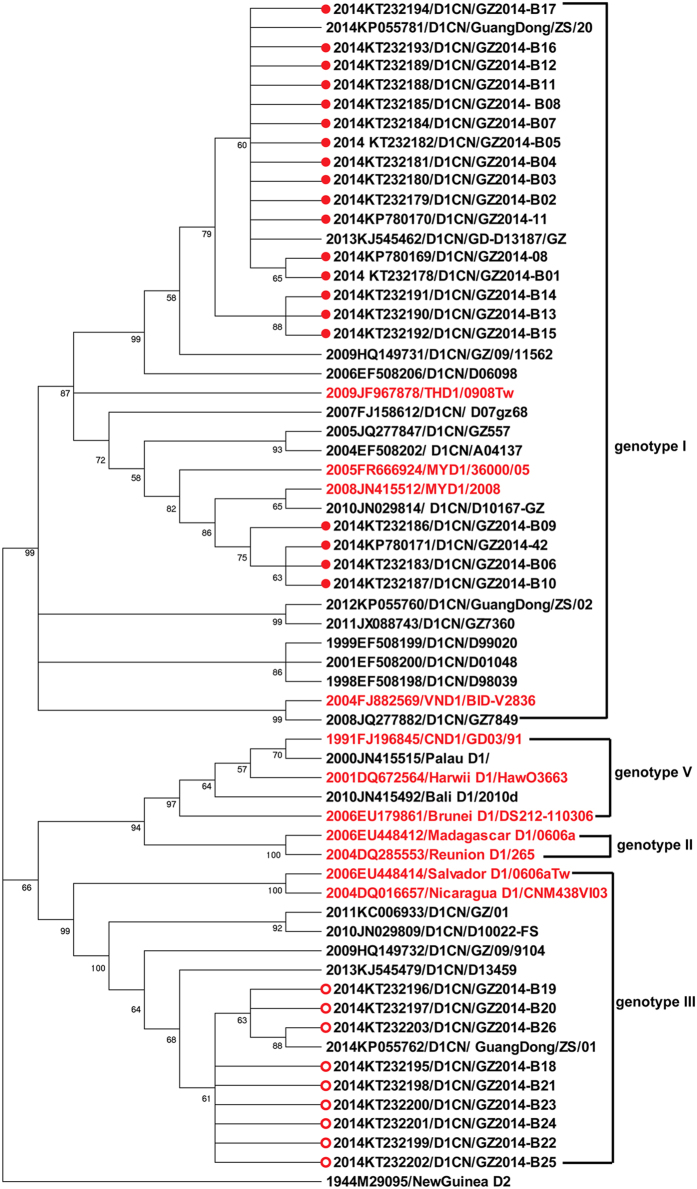
Phylogenetic analysis of isolated DENV-1 along with the homologous strains reported in Guangdong Province. A phylogenetic tree was constructed by the Neighbor-joining method based on the Tajima-Nei model using MEGA5.21 software. The bootstrap value was set for 1000 repetitions. Branches corresponding to partitions reproduced in fewer than 50% of the bootstrap replicates were collapsed. The percentages of replicate trees in which the associated dengue virus isolates clustered together in the bootstrap test (1000 replicates) are shown next to the branches. The D2 strain of New Guinea reported in 1944 (M29095) was used as an out-group to root the tree. The sequences in red are references for different genotypes (DENV-1 Genotype I: JN415512, FR666924, FJ882569 and JF967878; DENV-1 Genotype V: DQ672564, FJ196845, and EU179861; DENV-1 Genotype III: EU448414 and DQ016657; DENV-1 Genotype II: EU448412 and DQ285553). The strains isolated in this outbreak that were classified as Genotype I are labeled with red dots, and those classified as genotype II are labeled with red circles.

Among the 29 strains of DENV-1, 20 belong to Genotype I and share more than 98% identity with each other, and 9 are Genotype III and share more than 99% identity with each other; these two genotypes exhibit only approximately 90% identity (Fig. 3). Among the 4 strains of DENV-2, 3 share approximately 98% identity with each other and are of the Cosmopolitan Genotype; this clade shares only approximately 90% identity with the other strain (GZ2014-B28), which was identified as Asian I Genotype (Fig. 3).

**Figure 4:**
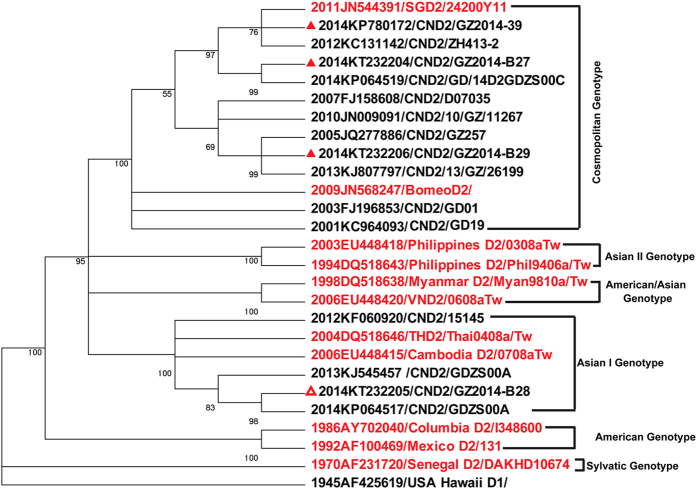
Phylogenetic analysis of isolated DENV-2 along with the homologous strains reported in Guangdong Province. A phylogenetic tree was constructed by the Neighbor-joining method based on the Tajima-Nei model by using MEGA5.21 software. The bootstrap value was set for 1000 repetitions. Branches corresponding to partitions reproduced in fewer than 50% of the bootstrap replicates were collapsed. The percentages of replicate trees in which the associated dengue virus isolates clustered together in the bootstrap test (1000 replicates) are shown next to the branches. The D1 strain of USA Hawaii reported in 1945 (AF425619) was used as an out-group to root the tree. The sequences in red are references for different genotypes (DENV-2 Cosmopolitan genotype: JN544391 and JN568247; Asian I Genotype: DQ518646 and EU448415; Asian II Genotype: EU448418 and DQ518643; American/Asian Genotype: DQ518638 and EU448420; American Genotype: AY702040 and AF100469). The strains of GZ2014-B27, GZ2014-B29, and GZ2014-39 isolated in this outbreak classified as the Cosmopolitan Genotype are labeled with red triangles. GZ2014-B28 was classified as Asian I Genotype, and it is labeled with a red hollow triangle.

Strains 2014-B01 (gb|KT232204), 2014-B26 (gb |KT232203), 2014-B27 (gb| KT232205), and GZ2014-B28(gb| KT232204) were used as the representative strains of DENV-1 Genotype I, DENV-1 Genotype III, DENV-2 Cosmopolitan Genotype, and DENV-2 Asian I Genotype, respectively, for blast searches of homologous sequences in GenBank. The representative homologous sequences reported in each year in Guangdong were selected for generating a phylogenetic tree. The phylogenetic analysis showed that DENV-1 Genotype I was reported in Guangdong Province in 1998, 1999, and 2001 and then continuously during 2004–2014 (Fig. 3); these sequences share 96.5–99.9% identity with each other (Supplementary Table 1). This evidence shows that this Genotype had been stably sustained in Guangdong Province. DENV-1 Genotype III has been reported in Guangdong since 2009, except for 2012 (Fig. 3), and these sequences share approximately 99% identity with each other (Supplementary Table 2). DENV-2 Cosmopolitan Genotype and Asian I Genotype have also been stably sustained in Guangdong since 2012 (Fig. 4), and the sequences shared approximately 99% identity within each genotype (Supplementary Tables 4 and 5).

**Figure 5:**
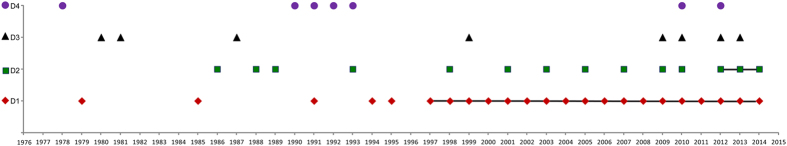
Serotypes of dengue virus reported in Guangdong Province. (1997–2006 data are from reference [9], 2005–2012 data are from reference [11], except that the DENV-1 1998, 2013, 2014 and DENV-2 2003, 2007, 2013, 2014 data are from GenBank). DENV-1 serotype strains were continuously reported from 1997 to 2014, and DENV-2 serotype strains were reported year by year from 2012 to 2014.

In addition, all the serotypes of dengue virus detected in Guangdong Province in every year were reviewed, and the data are shown in Fig. 5. In Guangdong, DENV-1 has been continuously reported since 1997 and DENV-2 has been stably reported since 2012.

### Vector surveillance and control measures

**Figure 6:**
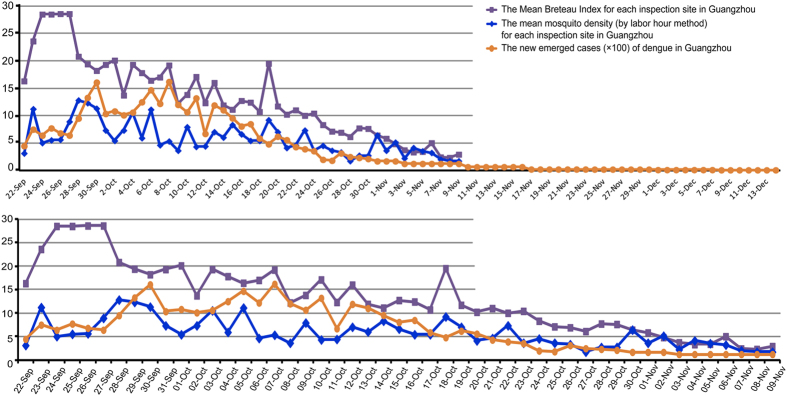
The relationship between daily newly emerged cases of dengue and the mosquito density in Guangzhou City in 2014. In this large outbreak of dengue in Guangdong Province, Guangzhou City suffered the most, as it was the major epidemic area. The daily newly emerged cases of dengue reached a peak between September 29 and October 14, exceeding 1000 cases each day, and then decreased quickly after October 14, from 1074 cases per day to only 24 cases per week from December 8 to 14. The mean Breteau Index in the different districts of Guangzhou City decreased quickly after September 27, dropped from 28.6 to approximately 5 on November 1 and was stably maintained at 5 or less thereafter. The mosquito density evaluated by the labor hour method showed a similar declining trend. The daily new cases decreased dramatically after October 13, approximately 20 days after the implementation of large-scale mosquito control. The declining trend of daily new cases also showed a 20-day lag in comparison with the mosquito density curve.

Since the first indigenous case reported in Guangdong Province in mid-June of 2014, the agencies responsible for vector control have paid great attention to mosquito density surveillance and mosquito eradication. At the same time, other measures were also taken to control dengue expansion, such as increasing the awareness of medical clinic personnel to take note of fever cases for early diagnosis and the isolation of dengue patients and also publicizing dengue prevention through all types of media. The mosquito eradication methods included traditional chemical insecticide spraying and the removal of pooled water in such receptacles as vases, tires, and tree holes in the neighborhood. When the newly emerged cases increased on a large scale in mid-August, the government started to take a role in the organization of mosquito control, such as spraying with chemical insecticides in parks or mountains and releasing *Gambusia affinis* in ponds. These measures were largely effective in mosquito control. In Guangzhou City, which suffered the most during this dengue outbreak, the mean Breteau Index (BI) in the different districts showed an apparent decline after September 27, from 28.6 to approximately 5 on November 1, and was stably maintained at 5 or less ever since. Additionally, the mosquito density evaluated by the labor hour method also showed a declining trend that was similar to the BI trend. The daily new cases of dengue in Guangzhou City peaked between September 29 and October 14 and declined dramatically thereafter (Fig. 6), with the peak of daily new cases lagging by approximately 20 days compared to the peak of mosquito density (Fig. 6).

## Discussion

In this large outbreak of dengue in Guangdong Province, multiple sources of infection were identified in either the epidemiological analysis or by the phylogenetic analysis of the virus. As early as June 11, 2014, Guangdong had reported 11 imported dengue cases and 1 indigenous case. From this outbreak, we successfully isolated 33 DENV strains from the serum of acute-stage patients, including 20 of DENV-1 Genotype I, 9 of DENV-1 Genotype III, 3 of DENV-2 Cosmopolitan Genotype, and 1 of DENV-2 Asian I Genotype. The DENV-1 Genotype I isolates appeared to originate in Guangdong Province, as they were highly homologous, with more than 96.6% identity in the E protein gene; these strains have been reported from Guangdong Province continuously since 2004 (Supplementary Table 1). The DENV-1 Genotype III strains isolated in 2014 were closely related to the strains reported from Guangdong since 2009, though no record was found in 2012, with more than 98.72% identity in the E protein gene (Supplementary Table 2). Furthermore, the DENV-2 Cosmopolitan Genotype and DENV-2 Asian I Genotype strains isolated in 2014 were also closely related to the viruses reported from Guangdong since 2012, with more than 97.5% and 97.85% identity, respectively, with strains reported in previous years. A review of the serotypes of DENV isolated from Guangdong Province also showed that DENV-1 had been continuously reported since 1997 and that DENV-2 had been stably reported since 2012 (Fig. 5). Such evidence indicates the possibility of dengue endemicity in Guangdong Province, which was also observed previously by Guo RN *et al.* when analyzing the prevalence and endemic nature of dengue infections in Guangdong^11^. The notion that dengue fever is an imported epidemic disease has also been challenged by the fact of the stable circulation of DENV-2 strains in Guangdong since their introduction in the 2000s^16^.

A dengue epidemic area is characterized by an epidemic of one or multiple serotypes but with no single virus being sustained^11^. According to the analysis of the prevalence and endemic nature of dengue infections in Guangdong during 2005-2011, only 9.89% of the cases were imported, whereas 90.11% were indigenous^11^. Indeed, the dengue epidemic in Guangdong is increasingly exhibiting the features of an endemic area, as suggested by the following: sustained virus, co-existence of multiple serotypes in the local area and a higher risk of infection in younger age groups^11^. These features were also identified in the large outbreak of dengue in 2014. Nonetheless, the confirmation of dengue endemicity in Guangdong may require more observation with regard to the virus resulted in the cases yearly in the future and find evidence in nature that DENV can be passed through mosquito eggs to survive through the winter and be sustained in adults after hatching, eventually causing new cases.

According to data from the Health Department of Guangdong Province, the dengue cases in Guangdong increased explosively from 2011 to 2014: 49 cases in 2011, 474 cases in 2012, 2894 cases in 2013, and more than 45,171 cases in 2014, the most in Guangdong history. Some factors present in Guangdong may have contributed to this large outbreak of dengue in 2014. First, the pandemic of dengue in countries of Southeast Asia has resulted in more imported sources of infection. Second, the high temperature and drenching rain result in the supernormal breeding of mosquitoes. According to the “2014 Climate Bulletin of Guangdong Province”, the average temperature in summer/autumn (June to November) in 2014 reached a record high temperature of 26.6 ^o^C, which was 0.8 ^o^C higher than the perennial summer/autumn temperature^17^. In addition, according to this report, although the average annual rainfall of 1652.5 mm in 2014 was not the highest on record, it was nonetheless at a very high level^17^. Third, the neglect of dengue infection at the early stage of the disease has hindered the treatment and isolation of patients and therefore disease prevention. Fourth, Guangdong is characterized by the most rapid urbanization in southern China, and the residents are highly concentrated and mobile, increasing the possibility of dengue transmission^15^; indeed, urbanization increases the density, larval development rate, and adult survival of *Ae. albopictus*, which in turn increases the ability of this mosquito to transmit dengue^15^.

In Guangdong Province, *Ae. albopictus*, a peridomestic mosquito, is the major vector for dengue rather than the fully domestic *Ae. aegypti*^9^. With global warming, elevated temperatures will reduce the extrinsic incubation period of *Aedes* vectors, promote vegetation growth that is favorable for mosquito development, and elevate rainfall, all promoting *Aedes* breeding^12^,^13^,^18^,^19^. The establishment of an early warning system to reinforce case management and medical treatment will help to control and prevent dengue in Guangdong, China^20^,^21^, and enhancing control measures to maintain *Aedes* breeding at a safe density level (BI < 5) is also important for dengue prevention^22^. More frequent BI investigation and intensive mosquito eradication is urgently needed in Guangdong Province to prevent dengue future outbreaks.

## Methods

### Ethics statement

As this research involved human blood, the aims of our study were explained to and written informed consent was obtained from all dengue patients involved in our study. For patients under 16 years old, consent from the parent or guardian was obtained. The study methods were reviewed and approved by the Institutional Ethics Review Board of Southern Medical University and were carried out in accordance with the approved guidelines. Data analysis was performed on anonymous datasets preserved at Nanfang Hospital and Zhongshan Boai Hospital affiliated with Southern Medical University and Guangzhou Eighth People’s Hospital.

### Data sources

Dengue is a notifiable disease in China. Hospitals and clinics report clinically diagnosed and laboratory-confirmed cases of dengue first to the local county or city Center of Disease Control and Prevention (CDC) and then to the provincial CDC. In the early stage of the outbreak in 2014, the Guangdong provincial CDC organized and performed laboratory verification, source tracing, and epidemiological investigation, such as searching for other dengue cases or clusters. As the outbreak expanded quickly to more than 1000 new cases every day by the end of September, the final diagnosis and hospitalization of new cases had to be undertaken by the local hospitals and reported to the provincial CDC. The case information in this study was retrieved anonymously from datasets preserved at Nanfang Hospital (located in Guangzhou city) and Zhongshan Boai Hospital (located in Zhongshan city) affiliated with Southern Medical University and Guangzhou Eighth People’s Hospital (located in Guangzhou City). The patients’ acute serum (collected within 1–5 days of illness onset) was also obtained from these hospitals. The dengue case numbers for each day of the outbreak were downloaded from the website of the Health Department of Guangdong Province (http://www.gdwst.gov.cn/).

### Case definitions and dengue infection diagnosis

Dengue fever (DF) diagnosis was made following the “national diagnostic criteria and principle of management of dengue fever (WS 216–2008)” and the “Guangdong Province guidelines for dengue fever diagnosis and treatment (2014)”.

Clinical presentation of DF includes acute onset of fever, severe headache, orbital pain, myalgia, arthralgia, fatigue, flush, rash, conjunctival congestion, positive tourniquet test, bleeding, liver enlargement, increase in hematocrit, fluid accumulation, and lethargy. A suspected case of DF is defined by DF symptoms and living in or traveling to an endemic region within 14 days before illness onset or presenting with leucopenia (white blood cell count < 4 × 10^9^/L) and thrombocytopenia (platelet count < 100 × 10^9^/L) at diagnosis. A clinically diagnosed DF case is defined by presenting with DF symptoms, living in or traveling to an endemic region within 14 days before disease onset, presenting with symptoms of leucopenia and thrombocytopenia, and having a positive result for anti-dengue virus IgM antibodies. A laboratory-confirmed case constitutes the detection of the virus non-structural protein 1 (NS1) antigen or ribonucleic acid (RNA) in the acute serum of a suspected case; if the virus is isolated from the patient’s blood, tissue or cerebrospinal fluid; or if the IgG titer in the recovery phase is 4 times higher than that in the acute phase.

Acute-phase sera (within 5 days of onset) were collected in the clinic for serum antibody, NS1 antigen and RNA detection. The detailed methods are as follows. Recovery-phase (15 days of onset) sera were collected. For real-time PCR diagnosis, the TaKaRa One Step Prime Script TM reverse-transcriptase polymerase chain reaction (RT-PCR) kit (Perfect Real Time) was used. For immuno-diagnosis, a dengue immunoglobulin M and G (IgM/IgG) enzyme-linked immunoassay kit (Zhong-shan Biological Engineering Co., Ltd., China) was used. The Panbio Early ELISA kit (Panbio Diagnostics, Brisbane, Australia) was used for NS1-based diagnostic testing of early infection of dengue.

### Dengue virus isolation

The acute serum from patients was diluted 1:10 with Dulbecco’s minimum essential medium (DMEM) (Life Technologies, USA) and used to inoculate baby hamster kidney (BHK-21) cells for 2 h. The infected cells were maintained in DMEM supplemented with 2% fetal bovine serum (Life Technologies) at 37 °C in 5% CO_2_. When complete cytopathic effects (CPE) were observed, the culture supernatant was collected and stored at −70 °C until use.

### Serotype identification and amplification of the E protein gene

A QIAamp Viral RNA Kit (Qiagen, 52906) was used to extract total RNA from the supernatant of the DENV-infected BHK-21 cell culture. A GoScript™ Reverse Transcription System (Promega, A5001) was used for the transcription of DENV RNA. The transcription and serotype identification primers were synthesized according to the report of Lanciotti RS^23^. E protein cDNA was amplified with the following primers: TypeI EF, ATGCGATGCGTGGGAATAGG; TypeI ER, CGCCTGAACCATGACTCCTA; TypeII EF, ATGCGTTGTATAGGAATATC; and TypeII ER, CCATTATTTCAAAAGGGATC. The E protein gene was amplified with Pfu DNA polymerase (Promega; cat. M7741) and cloned into the pEASY-Blunt plasmid (TransGen, CB101) for sequencing.

The diagnosis criteria of abnormal liver function caused by dengue infection are as follows: serum alanine transaminase (ALT) > 40 U/L and serum aspartate transaminase (AST) > 35 U/L.

### Phylogenetic analysis of isolated dengue viruses and homologous strains reported in Guangdong and retrospective review for serotypes of dengue virus reported in Guangdong

All E protein genes of the isolated virus were analyzed for identity using Clustal W, and blast searching was performed for homology to identify serotypes. The homologous sequences reported in Guangdong Province were downloaded and kept for identity comparison and phylogenetic analysis. The reference sequences were selected on the basis of the following inclusion criteria: 1) the sequence had to have an unambiguous serotype assignment and be classified as non-recombinant; and 2) the city/state of origin and sampling date were clear.

A phylogenetic analysis was performed to build two trees for the DENV-1 and DENV-2 datasets. The dengue virus genotype was analyzed by including the sequences with a known genotype when drawing the phylogenetic tree (DENV-1 Genotype I: JN415512, FR666924, FJ882569 and JF967878; DENV-1 Genotype V: DQ672564, FJ196845, and EU179861; DENV-1 Genotype III: EU448414 and DQ016657; DENV-1 Genotype II: EU448412 and DQ285553. DENV-2 Cosmopolitan genotype: JN544391 and JN568247; Asian II Genotype: EU448418 and DQ518643; American/Asian Genotype: DQ518638 and EU448420; Asian I Genotype: DQ518646 and EU448415; American Genotype: AY702040 and AF100469)^24^,^25^. The serotypes of dengue virus reported in Guangdong Province were reviewed with the published literature using GenBank as a supplement.

All the sequences in each group of DENV-1 and DENV-2 were aligned with the Clustal X algorithm^26^, and gaps were manually deleted while editing; positions containing gaps were removed from the final alignment. Phylogenetic trees were drawn using the Tajima-Nei model with the Neighbor-joining method and Mega5 software. The statistical robustness and reliability of the branching order within each phylogenetic tree were confirmed with a bootstrap analysis using 1000 replicates. Branches corresponding to partitions reproduced in fewer than 50% of the bootstrap replicates were collapsed. The percentages of replicate trees in which the associated dengue virus isolates clustered together in the bootstrap test (1000 replicates) are shown next to the branches.

In drawing the DENV-1 tree, 29 strains of serotype I isolated in this outbreak, GZ2014-08 (gb|KP780169), GZ2014-11 (gb|KP780170), GZ2014-42 (gb|KP780171), and GZ2014-B01~26 (gb|KT232178- KT232203), plus 19 strains of homologous sequences reported from Guangdong in previous years and the 11 DENV1 genotype markers were included. The D2 strain of New Guinea reported in 1944 (M29095) was used as an out-group to root the tree.

In drawing the DENV-2 tree, 4 strains of serotype II isolated in this outbreak, GZ2014-39 (gb|KP780172), and GZ2014-B27-29 (gb|KT232204-KT232206), plus 11 strains of homologous sequences reported from Guangdong in previous years and the 11 genotype markers were included. The D1 strain of USA Hawaii reported in 1945 (AF425619) was used as an out-group to root the tree.

### Dengue vector monitoring and control

Vector monitoring is fulfilled by the local CDC and usually performed monthly to investigate the mosquito density according to the Breteau Index (BI) (number of positive containers for *Aedes* per 100 houses) for larvae^22^ and labor hours (number of mosquitoes caught with an electronic mosquito collection machine in an area of 15 m^2^ in 15 minutes continuously, 4 times at 1 h after sunset) for adult mosquitoes^27^. The mosquito density in one surveillance location is the mean number of mosquitoes caught at 5 different sites at this location. *Aedes albopictus* density information was retrieved from Guangzhou mosquito vector density monitoring that is released daily by the Guangzhou City CDC. During the dengue outbreak, the staff from the CDC, the health department and each community implemented mosquito investigation and offered instruction for dengue prevention and mosquito eradication in residents’ homes and neighborhoods. Large-scale mosquito control, which included fogging with insecticide, releasing *Gambusia affinis* in ponds and cleaning out the breeding habitats of *Ae. albopictus*, was organized by the government.

## Additional Information

**How to cite this article**: Shen, S.-Q. *et al.* Multiple Sources of Infection and Potential Endemic Characteristics of the Large Outbreak of Dengue in Guangdong in 2014. *Sci. Rep.* **5**, 16913; doi: 10.1038/srep16913 (2015).

## Supplementary Material

Supplementary data

## Figures and Tables

**Table 1:** Characteristics of 200 clinically diagnosed cases and 2050 laboratory-confirmed cases of dengue fever from the three hospitals in Guangdong Province.

Symptoms	Number of cases	Proportion (%)	Diagnosis methods
Fever	200	100	200 clinically diagnosed cases
Headache	70	35.0	
Rash	59	29.5	
Myalgia	121	60.5	
bleeding spots under the skin)	40	20.0	
Bleeding	14	7.0	
Arthralgia	36	18.0	
Fever + Headache + Rash + Myalgia	13.8	6.9	
Leucopenia	1152	56.2	2050 laboratory-confirmed cases
Thrombocytopenia	1259	61.4	
*Serum AST elevation	1574	76.8	
*Serum ALT elevation	873	42.6	

^*^Serum AST and ALT levels were not included in the Dengue diagnosis standard, but were used as indicators to show liver abnormality.
