# Supplementary material for: Multiple Sources of Infection and Potential Endemic Characteristics of the Large Outbreak of Dengue in Guangdong in 2014
Source: Sci Rep. 2015 Nov 23;5:16913. doi: 10.1038/srep16913 (PMC4655357; doi:10.1038/srep16913)
Supplement: Supplementary data [file srep16913-s1.doc]

**Multiple Sources of Infection and Potential Endemic Characteristics of the Large Outbreak of Dengue in Guangdong in 2014**

Shu-Qun Shen ^1#^, Hai-Xia Wei ^1#^, Yong-Hang Fu^2^, Hao Zhang^3^, Qing-Yi Mo^4^, Xiao-Jun Wang^1^, Sheng-Qun Deng^1^, Wei Zhao^1^, Yu Liu^1^, Xiao-Shuang Feng^1^, Wei Chen^5^, Hong-Juan Peng^*1^

1. Guangdong Provincial Key Laboratory of Tropical Disease Research, and Key Laboratory of Prevention and Control for Emerging Infectious Diseases of Guangdong Higher Institutes, School of Public Health and Tropical Medicine, Southern Medical University, #1023 South Shatai Road, Guangzhou, Guangdong Province, 510515, China.
2. Department of Clinical Laboratory, the 458th Hospital of PLA, #801 Dongfeng Dong Road, Guangzhou, Guangdong Province, 510602, China.
3. Guangdong Provincial Key Laboratory of Viral Hepatitis Research, Department of Infectious Diseases, Nanfang Hospital, Southern Medical University.
4. Department of pediatrics, Zhongshan Boai Hospital Affiliated to Southern Medical University, Zhongshan, Guangdong Province, 528403, China.
5. Max-Delbrueck-Center for Molecular Medicine. Robert-Roessle-str. 10, Berlin, 13125, Germany

^#^ These two authors contributed equally to this work

* Corresponding author

E-mail: [hongjuan@smu.edu.cn](mailto:hongjuan@smu.edu.cn)

**Supplementary data**

**S. Table 1 The sequence identity between the DENV1-Genotype I** strains reported in Guangdong province

| **Identity (%)** | **1-2014**  **KT232178** | **2-2014**  **KP055781** | **3-2013**  **KJ545462** | **4-2012**  **KP055760** | **5-2011**  **JX088743** | **6-2010**  **JN029814** | **7-2009**  **HQ149731** | **8-2008**  **JQ277882** | **9-2007**  **FJ158612** | **10-2006**  **EF508206** | **11-2005**  **JQ277847** | **12-2004**  **EF508202** | **13-2001**  **EF508200** | **14-1999**  **EF508199** |
| --- | --- | --- | --- | --- | --- | --- | --- | --- | --- | --- | --- | --- | --- | --- |
| **2-2014**  **KP055781** | **99.93** |  |  |  |  |  |  |  |  |  |  |  |  |  |
| **3-2013**  **KJ545462** | **99.80** | **99.87** |  |  |  |  |  |  |  |  |  |  |  |  |
| **4-2012**  **KP055760** | **97.44** | **97.51** | **97.51** |  |  |  |  |  |  |  |  |  |  |  |
| **5-2011**  **JX088743** | **97.43** | **97.5** | **97.5** | **99.53** |  |  |  |  |  |  |  |  |  |  |
| **6-2010**  **JN029814** | **98.11** | **98.18** | **98.18** | **97.85** | **97.84** |  |  |  |  |  |  |  |  |  |
| **7-2009**  **HQ149731** | **99.46** | **99.53** | **99.53** | **97.85** | **97.84** | **98.52** |  |  |  |  |  |  |  |  |
| **8-2008**  **JQ277882** | **96.55** | **96.62** | **96.62** | **97.03** | **97.23** | **96.69** | **96.96** |  |  |  |  |  |  |  |
| **9-2007**  **FJ158612** | **98.25** | **98.32** | **98.32** | **97.71** | **97.77** | **98.92** | **98.65** | **96.75** |  |  |  |  |  |  |
| **10-2006**  **EF508206** | **99.26** | **99.33** | **99.33** | **98.05** | **98.04** | **98.86** | **99.66** | **97.03** | **98.99** |  |  |  |  |  |
| **11-2005**  **JQ277847** | **98.51** | **98.58** | **98.58** | **98.17** | **98.11** | **99.19** | **98.92** | **96.96** | **99.26** | **99.26** |  |  |  |  |
| **12-2004**  **EF508202** | **98.38** | **98.45** | **98.45** | **97.98** | **97.97** | **99.06** | **98.79** | **96.82** | **99.06** | **99.12** | **99.73** |  |  |  |
| **13-2001**  **EF508200** | **97.98** | **98.05** | **98.05** | **98.38** | **98.51** | **98.52** | **98.38** | **97.36** | **98.52** | **98.72** | **98.78** | **98.65** |  |  |
| **14-1999**  **EF508199** | **98.11** | **98.18** | **98.18** | **98.65** | **98.78** | **98.79** | **98.52** | **97.63** | **98.52** | **98.86** | **98.92** | **98.79** | **99.33** |  |
| **15-1998**  **EF508198** | **98.25** | **98.32** | **98.32** | **98.79** | **98.92** | **98.79** | **98.65** | **97.77** | **98.65** | **98.99** | **99.05** | **98.92** | **99.46** | **99.73** |

**S. Table 2 The sequence identity between the DENV1-Genotype III strains reported in Guangdong province**

| **Identity (%)** | **1-2014**  **KT232203** | **2-2014**  **KP055762** | **3-2013**  **KP685234** | **4-2011**  **KC006933** | **5-2010**  **JN029809** |
| --- | --- | --- | --- | --- | --- |
| **2-2014**  **KP055762** | **99.93** |  |  |  |  |
| **3-2013**  **KP685234** | **99.60** | **99.66** |  |  |  |
| **4-2011**  **KC006933** | **98.72** | **98.72** | **99.06** |  |  |
| **5-2010**  **JN029809** | **99.06** | **99.12** | **99.46** | **99.46** |  |
| **6-2009**  **HQ149732** | **99.53** | **99.6** | **99.93** | **99.53** | **99.53** |

**S. Table 3 The sequence identity between the DENV2-Cosmopolitan Genotype strains reported in Guangdong province**

| **Identity (%)** | **1-2014**  **KT232204** | **2-2014**  **KP064519** | **3-2013**  **KJ807797** | **4-2012**  **KC131142** | **5-2010**  **JN009091** | **6-2007**  **FJ158608** | **7-2005**  **JQ277886** | **8-2003**  **FJ196853** |
| --- | --- | --- | --- | --- | --- | --- | --- | --- |
| **2-2014**  **KP064519** | **100.0** |  |  |  |  |  |  |  |
| **3-2013**  **KJ807797** | **97.45** | **97.31** |  |  |  |  |  |  |
| **4-2012**  **KC131142** | **99.12** | **98.99** | **97.17** |  |  |  |  |  |
| **5-2010**  **JN009091** | **98.33** | **97.98** | **98.05** | **97.91** |  |  |  |  |
| **6-2007**  **FJ158608** | **98.14** | **97.98** | **97.85** | **97.91** | **98.59** |  |  |  |
| **7-2005**  **JQ277886** | **98.04** | **97.85** | **99.26** | **97.78** | **98.65** | **98.45** |  |  |
| **8-2003**  **FJ196853** | **98.33** | **98.38** | **97.51** | **98.32** | **97.98** | **97.91** | **97.98** |  |
| **9-2001**  **KC964093** | **97.45** | **97.58** | **96.3** | **97.24** | **96.9** | **96.84** | **96.9** | **98.32** |

**S. Table 4 The sequence identity between the DENV2-Asian I Genotype strains reported in Guangdong province**

| **Identity (%)** | **1-2014**  **KT232205** | **2-2014**  **KP064517** | **3-2013**  **KJ545457** |
| --- | --- | --- | --- |
| **2-2014**  **KP064517** | **99.71** |  |  |
| **3-2013**  **KJ545457** | **98.63** | **98.92** |  |
| **4-2012**  **KF060920/** | **97.85** | **98.25** | **98.65** |
